# Supplementary material for: Effects of inconsistent reporting, regulation changes and market demand on abundance indices of sharks caught by pelagic longliners off southern Africa
Source: PeerJ. 2018 Oct 24;6:e5726. doi: 10.7717/peerj.5726 (PMC6203943; doi:10.7717/peerj.5726)
Supplement: Table S1 [file peerj-06-5726-s002.docx]

|  |  | Total hooks | Blue shark catch (numbers) | Shortfin mako catch (numbers) |
| --- | --- | --- | --- | --- |
| Month | January | 1 714 538 | 18 575 | 29 569 |
|  | February | 1 684 870 | 17 866 | 31 051 |
|  | March | 2 292 331 | 34 150 | 41 778 |
|  | April | 3 562 462 | 28 080 | 35 649 |
|  | May | 5 250 171 | 29 648 | 34 946 |
|  | June | 6 656 354 | 33 034 | 42 360 |
|  | July | 7 518 715 | 28 196 | 26 358 |
|  | August | 6 748 990 | 18 116 | 22 155 |
|  | September | 6 212 049 | 16 939 | 17 396 |
|  | October | 4 796 017 | 22 458 | 19 362 |
|  | November | 3 190 558 | 27 009 | 33 193 |
|  | December | 2 368 882 | 27 991 | 36 222 |
| Year | 2000 | 1 309 472 | 1 207 | 2 331 |
|  | 2001 | 1 283 246 | 2 512 | 6 291 |
|  | 2002 | 2 213 491 | 3 580 | 3 889 |
|  | 2003 | 1 799 911 | 2 679 | 7 497 |
|  | 2004 | 2 774 544 | 13 189 | 7 534 |
|  | 2005 | 3 520 426 | 27 396 | 19 729 |
|  | 2006 | 1 377 984 | 19 695 | 11 602 |
|  | 2007 | 4 365 937 | 30 271 | 9 632 |
|  | 2008 | 3 972 649 | 26 217 | 19 777 |
|  | 2009 | 4 583 152 | 25 540 | 25 356 |
|  | 2010 | 5 047 126 | 15 816 | 11 271 |
|  | 2011 | 5 899 429 | 31 508 | 50 933 |
|  | 2012 | 4 040 415 | 13 930 | 27 483 |
|  | 2013 | 4 285 951 | 22 125 | 28 004 |
|  | 2014 | 2 891 314 | 27 335 | 76 155 |
|  | 2015 | 2 630 890 | 39 062 | 62 555 |
| ICCAT | W | 7 489 330 | 118 688 | 27 070 |
|  | SW | 4 975 343 | 182 113 | 95 917 |
| IOTC | S | 21 489 223 | 74 264 | 131 123 |
|  | E | 18 042 041 | 21 005 | 21 921 |
| Fleet | Local | 21 560 474 | 258 783 | 352 791 |
|  | Foreign | 30 435 463 | 43279 | 17248 |
