# Supplementary material for: Effects of inconsistent reporting, regulation changes and market demand on abundance indices of sharks caught by pelagic longliners off southern Africa
Source: PeerJ. 2018 Oct 24;6:e5726. doi: 10.7717/peerj.5726 (PMC6203943; doi:10.7717/peerj.5726)
Supplement: Table S2 — Included are the estimate, standard error, and p-values for each coefficient of the model. The variables Year, Month and Observer were included as fixed effects in the models. Vessel was the only random effect. The explanatory variables (1—Vessel) + Year + Month + Fleet + Observer consistently provided the lowest BIC for all the models. [file peerj-06-5726-s003.docx]

| West subregion | | | | | | | | | | | | |
| --- | --- | --- | --- | --- | --- | --- | --- | --- | --- | --- | --- | --- |
| Model | Blue shark CPUE | | | Blue shark CPUE | | | Mako CPUE | | | Mako CPUE | | |
| Explanatory variables | (1\|Vessel) + Year + Month + Fleet + Observer | | |  |  |  |  |  |  |  |  |  |
| Error | Binomial |  |  | Gamma |  |  | Binomial |  |  | Gamma |  |  |
| Link | Logit |  |  | Log |  |  | Logit |  |  | Log |  |  |
| BIC | 4 102 |  |  | 18 151 |  |  | 5 599 |  |  | 18 146 |  |  |
|  | Estimate | Std. Error | Pr(>\|z\|) | Estimate | Std. Error | Pr(>\|z\|) | Estimate | Std. Error | Pr(>\|z\|) | Estimate | Std. Error | Pr(>\|z\|) |
| 2000 | -1.6724 | 0.6411 | 0.0091 | 1.8840 | 0.3483 | 0.0000 | -0.9918 | 0.3670 | 0.0069 | 1.2749 | 0.1887 | 0.0000 |
| 2001 | -2.4627 | 0.6423 | 0.0001 | 2.1045 | 0.3536 | 0.0000 | -1.5258 | 0.3611 | 0.0000 | 0.9530 | 0.1862 | 0.0000 |
| 2002 | -1.0668 | 0.6225 | 0.0865 | 2.6114 | 0.3616 | 0.0000 | -1.9297 | 0.3600 | 0.0000 | 0.7403 | 0.1898 | 0.0001 |
| 2003 | -0.5088 | 0.6146 | 0.4078 | 3.0301 | 0.3509 | < 2e-16 | -1.0136 | 0.3456 | 0.0034 | 1.4554 | 0.1811 | 0.0000 |
| 2004 | 0.1797 | 0.6189 | 0.7716 | 3.2623 | 0.3454 | < 2e-16 | -0.5029 | 0.3405 | 0.1397 | 1.7417 | 0.1787 | < 2e-16 |
| 2005 | -0.7338 | 0.6448 | 0.2551 | 3.7329 | 0.3457 | < 2e-16 | -0.9734 | 0.3656 | 0.0078 | 1.5188 | 0.1960 | 0.0000 |
| 2006 | 0.0967 | 0.6887 | 0.8883 | 3.7050 | 0.3731 | < 2e-16 | -0.5618 | 0.4020 | 0.1623 | 1.7259 | 0.2093 | < 2e-16 |
| 2007 | -2.1923 | 0.6364 | 0.0006 | 2.4398 | 0.3431 | 0.0000 | -1.1820 | 0.3651 | 0.0012 | 1.5034 | 0.1966 | 0.0000 |
| 2008 | -2.2265 | 0.6571 | 0.0007 | 3.0659 | 0.3352 | < 2e-16 | -2.2261 | 0.3886 | 0.0000 | 1.1640 | 0.2035 | 0.0000 |
| 2009 | -2.4620 | 0.6647 | 0.0002 | 2.9467 | 0.3738 | 0.0000 | -0.9236 | 0.3860 | 0.0167 | 1.3129 | 0.2022 | 0.0000 |
| 2010 | -2.6038 | 0.6858 | 0.0001 | 4.1020 | 0.4152 | < 2e-16 | 0.2147 | 0.4022 | 0.5934 | 1.4203 | 0.1995 | 0.0000 |
| 2011 | 2.6493 | 0.6821 | 0.0001 | 5.3237 | 0.3621 | < 2e-16 | -0.2186 | 0.4016 | 0.5863 | 1.7939 | 0.2036 | < 2e-16 |
| 2012 | 2.4269 | 0.6991 | 0.0005 | 5.0944 | 0.3602 | < 2e-16 | 0.8705 | 0.4556 | 0.0561 | 1.6510 | 0.2031 | 0.0000 |
| 2013 | 1.9967 | 0.6714 | 0.0029 | 4.1723 | 0.3562 | < 2e-16 | 1.5285 | 0.4328 | 0.0004 | 1.7900 | 0.1979 | < 2e-16 |
| 2014 | 2.0787 | 0.6633 | 0.0017 | 4.5300 | 0.3540 | < 2e-16 | 1.5321 | 0.4111 | 0.0002 | 1.9277 | 0.1932 | < 2e-16 |
| 2015 | 1.6587 | 0.6178 | 0.0073 | 3.9258 | 0.3301 | < 2e-16 | 2.9440 | 0.4565 | 0.0000 | 2.1116 | 0.1754 | < 2e-16 |
| January | - | - | - | - | - | - | - | - | - | - | - | - |
| February | 0.3212 | 0.4531 | 0.4784 | 0.0043 | 0.1889 | 0.9819 | 0.4587 | 0.3205 | 0.1523 | -0.1606 | 0.1369 | 0.2407 |
| March | 1.9094 | 0.3519 | 0.0000 | -0.4396 | 0.1865 | 0.0184 | 0.2054 | 0.3081 | 0.5051 | 0.4465 | 0.1270 | 0.0004 |
| April | 1.1784 | 0.2826 | 0.0000 | -0.8726 | 0.1665 | 0.0000 | 0.1997 | 0.2489 | 0.4223 | 0.0555 | 0.1050 | 0.5975 |
| May | 0.9102 | 0.2412 | 0.0002 | -0.6700 | 0.1469 | 0.0000 | 0.3714 | 0.2097 | 0.0765 | 0.2156 | 0.0895 | 0.0160 |
| June | 0.5696 | 0.2295 | 0.0131 | -0.6550 | 0.1504 | 0.0000 | 0.3763 | 0.1948 | 0.0534 | -0.1163 | 0.0861 | 0.1770 |
| July | 0.6302 | 0.2291 | 0.0060 | -0.6321 | 0.1526 | 0.0000 | 0.5693 | 0.1942 | 0.0034 | -0.2343 | 0.0862 | 0.0065 |
| August | 0.9314 | 0.2393 | 0.0001 | -0.3559 | 0.1559 | 0.0224 | 0.7170 | 0.2039 | 0.0004 | 0.0862 | 0.0893 | 0.3346 |
| September | 1.0748 | 0.2587 | 0.0000 | -0.2686 | 0.1669 | 0.1076 | 0.5382 | 0.2181 | 0.0136 | 0.1183 | 0.0969 | 0.2224 |
| October | 0.4968 | 0.2601 | 0.0562 | -0.7184 | 0.1608 | 0.0000 | -0.1028 | 0.2158 | 0.6339 | -0.0690 | 0.0998 | 0.4892 |
| November | 0.3413 | 0.2649 | 0.1976 | -0.7061 | 0.1634 | 0.0000 | 0.2634 | 0.2173 | 0.2254 | -0.0048 | 0.0959 | 0.9597 |
| December | 0.3282 | 0.2625 | 0.2112 | -0.8124 | 0.1623 | 0.0000 | 0.3379 | 0.2286 | 0.1393 | -0.1654 | 0.0961 | 0.0853 |
| Fleet - Local | 0.0650 | 0.4278 | 0.8793 | 0.4064 | 0.3093 | 0.1888 | 0.5392 | 0.2128 | 0.0113 | -0.0078 | 0.1162 | 0.9468 |
| Fleet - Foreign | - | - | - | - | - | - | - | - | - | - | - | - |
| Observer - Unknown | - | - | - | - | - | - | - | - | - | - | - | - |
| Observer - No | -0.3864 | 0.1992 | 0.0525 | -0.3426 | 0.1041 | 0.0010 | 0.4909 | 0.1451 | 0.0007 | 0.1510 | 0.0757 | 0.0461 |
| Observer - Yes | -0.2058 | 0.2098 | 0.3267 | -0.4294 | 0.1158 | 0.0002 | 0.3239 | 0.1548 | 0.0364 | 0.0142 | 0.0804 | 0.8597 |
